# CircTBX5 knockdown modulates the miR-558/MyD88 axis to alleviate IL-1β-induced inflammation, apoptosis and extracellular matrix degradation in chondrocytes via inactivating the NF-κB signaling

**DOI:** 10.1186/s13018-023-03949-5

**Published:** 2023-07-01

**Authors:** Wei Wei, Hongjie Mu, Qiaoyi Cui, Peng Yu, Tong Liu, Tao Wang, Lin Sheng

**Affiliations:** 1 Department of Rehabilitation Medicine, Yantaishan Hospital, Yantai, China; 2 Foot and Ankle Surgery, Yantaishan Hospital, Yantai, China; 3 Sports Medicine, Yantaishan Hospital, Yantai, China; 4 Department of Medicine, Yantai City Yu Huangding Hospital, No. 20 Yuhuang East Road, Zhifu District, Yantai, 264000 Shandong China

**Keywords:** CircTBX5, Osteoarthritis, Chondrocyte, IL-1β, miR-558, MyD88

## Abstract

**Background:**

It has been widely shared that the dysregulation of circular RNA (circRNA) may contribute to the progression of osteoarthritis (OA). OA is characterized by persistent chondrocyte injury. We aimed to clarify the role of circTBX5 in IL-1β-induced chondrocyte injury.

**Methods:**

The expression of circTBX5, miR-558 and MyD88 mRNA was measured using quantitative real-time PCR (qPCR). Cell viability, proliferation and apoptosis were assessed by CCK-8, EdU or flow cytometry assay. The protein levels of extracellular matrix (ECM)-associated markers, MyD88, IkBα, p65 and phosphorylated IkBα were measured by western blot. The release of inflammatory factors was assessed by ELISA. The targets of circTBX5 were screened by RIP and pull-down assay. The putative binding between miR-558 and circTBX5 or MyD88 was validated by dual-luciferase reporter assay.

**Results:**

CircTBX5 and MyD88 were enhanced, while miR-558 was downregulated in OA cartilage tissues and IL-1β-treated C28/I2 cells. IL-1β induced C28/I2 cell injury by impairing cell viability and proliferation and promoting cell apoptosis, ECM degradation and inflammatory response, while circTBX5 knockdown alleviated IL-1β induced injury. CircTBX5 bound to miR-558 to regulate IL-1β induced cell injury. In addition, MyD88 was a target of miR-558, and circTBX5 targeted miR-558 to positively regulate MyD88 expression. MiR-558 enrichment attenuated IL-1β induced injury by sequestering MyD88 expression. Moreover, circTBX5 knockdown weakened the activity of NF-κB signaling, while miR-558 inhibition or MyD88 overexpression recovered the activity of NF-κB signaling.

**Conclusion:**

CircTBX5 knockdown modulated the miR-558/MyD88 axis to alleviate IL-1β induced chondrocyte apoptosis, ECM degradation and inflammation via inactivating the NF-кB signaling pathway.

**Supplementary Information:**

The online version contains supplementary material available at 10.1186/s13018-023-03949-5.

## Introduction

Osteoarthritis (OA) is the most common chronic joint disease, threatening millions of people worldwide [1]. The incidence of OA is increasing because of the aging of the population, and the prevalence of obesity and metabolic syndrome has also exacerbated the development of OA [2, 3]. OA mainly causes pain, stiffness, and mobility difficulties, leading to progressive disability [1]. In anatomical aspect, one of the salient features of OA is the progressive destruction of articular cartilage [4]. Chondrocytes are the main cell group in cartilage and play an important role in the homeostasis of cartilage metabolism [5]. Healthy articular chondrocytes are usually static, highly differentiated cells, and they maintain a flexible extracellular matrix (ECM) through the balance between the anabolism and catabolism of matrix components [6]. Therefore, exploring the molecular mechanisms of chondrocyte dysfunction is crucial for understanding the pathogenesis of OA.

Non-coding RNA, as important regulators in physiopathology, diagnosis and therapeutic potential, have been broadly exploited in human diseases [7–10]. CircRNA, as a new class of non-coding RNAs, is attracting extensive attention from researchers. Just as its name implies, circRNA is unique in its circular closed structure, with no 3′ cap and 5′ tail. CircRNA is produced from precursor through a “back-splicing” mechanism, and it harbors higher stability than its parental gene. By regulating host genes, functioning as molecular sponges of microRNAs (miRNAs) or interacting with RNA binding proteins (RBPs), circRNAs are implicated in a variety of biological processes and serve as important regulators in multiple human diseases and cancers. The results of circRNA expression profile acquired from sequencing technology identify numerous new circRNAs that are differently expressed between OA patients and normal controls [11, 12]. Progressive studies have discussed the protective or accelerative role of several circRNAs on chondrocyte dysfunction [13, 14], providing the molecular basis for circRNA to be involved in OA development. IL-1β is a major cytokine in proinflammatory signaling pathways and is widely used to treat chondrocytes to construct a pathological condition of OA. For example, circ_0114876 downregulation ameliorated IL-1β-induced chondrocyte apoptosis and inflammation [15]. A previous study performed circRNA microarray analysis using chondrocyte samples of knee joint from OA patients and non-OA patients, and we observed that circ_0003176 was aberrantly upregulated in OA chondrocytes [16]. Circ_0003176 is derived from T-box transcription factor 5 (TBX5) gene, also named circTBX5. However, the role of circTBX5 in OA is unclear, and its functional effects in OA chondrocytes have not been explored.

The public tools, such as circular RNA Interactome and circBank, are widely applied to computationally predict miRNAs targeted by certain circRNA [17, 18]. Intriguingly, miR-558 is putatively identified as a target of circTBX5. A previous study reported that miR-558 expression was remarkably declined in OA cartilage tissues and IL-1β-administered chondrocytes, and miR-558 affected cartilage homeostasis [19], which strongly evidenced that miR-558 was involved in OA progression. Nevertheless, it is unclear whether miR-558 is targeted by circTBX5 in regulating chondrocyte dysfunction.

It is universally acknowledged that miRNA governs gene expression via targeting gene 3′UTR. Whereupon, bioinformatics tool, Targetscan [20], is used to predict the target genes of miR-558. As predicted, miR-558 binds to MyD88 3′UTR. MyD88 was previously exhibited to be upregulated in chondrocytes with IL-1β stimulation, and the implication of MyD88 in chondrocyte dysfunction was widely reported [21, 22]. However, the interaction between miR-558 and MyD88 on chondrocyte dysfunction in OA progression has not been verified.

Our current study mainly investigated the functional effects of circTBX5 in IL-1β-treated human chondrocytes. Besides, we validated the binding relationship between miR-558 and circTBX5 or MyD88 and performed rescue experiments to explore their interactions on chondrocyte dysfunctions. Our study was the first to clarify the role and potential regulatory mechanism of circTBX5 in IL-1β-treated human chondrocytes, so as to provide the basis for circTBX5 in OA treatment.

## Materials and methods

### Clinical specimens

Cartilage tissues were obtained from patients with OA (n = 20; during knee replacement surgery; 10 males and 10 females; age: from 46 to 75 years) and subjects suffered from traffic accidents (Control; n = 20; during traumatic amputation surgery; 10 males and 10 females; age: from 48 to 73 years). These patients were surgically treated at Yantaishan Hospital. Written informed consent was obtained from all patients or guardians with their approval for study. Cartilage tissues were stored at − 80 °C conditions so as to subsequent use. This study was implemented with the permission of the Ethics Committee of Yantaishan Hospital.

### Cell treatment

Human chondrocyte, C28/I2, was purchased from TopBiotech (ShenZhen, China) and cultured in DMEM containing 10% FBS at 37 °C conditions with 5% CO_2_. IL-1β (Beyotime, Shanghai, China) was dissolved in sterile distilled water and stored at − 20 °C.

IL-1β for further use was diluted with cell culture medium at 10 ng/mL, and C28/I2 cells were treated with IL-1β for 24 h.

### Cell transfection

We customized siRNA targeting circTBX5 (si-circTBX5) and matched negative control (si-NC) from Geneseed (Guangzhou, China). The mimic of miR-558 (miR-558), the inhibitor of miR-558 (in-miR-558) and their controls (miR-NC and in-miR-NC) were bought from Ribobio (Guangzhou, China). Besides, MyD88 was overexpressed with the use of pcDNA vector. MyD88 overexpression vector (MyD88) and pcDNA vector were provided by Ribobio. Cells were cultured in 24-well plates overnight until 50% confluence, and cells were next transfected with these projects using Lipofectamine 3000 Reagent (Invitrogen, Carlsbad, CA, USA).

### Quantitative real-time PCR (qPCR)

Utilizing TRIzol Reagent (Beyotime), total RNA was isolated and then identified by a micro-spectrophotometer (Thermo Fisher, Waltham, MA, USA). RNA was assembled into cDNA using SuperScript™ III First-Strand Synthesis System (Thermo Fisher) or using miRNA First Strand cDNA Synthesis Kit (TianGen, Beijing, China), followed by qPCR using SYBR GreenER™ qPCR SuperMix (Invitrogen). Internal references used here were GAPDH and U6. The 2^−△△Ct^ method was employed to calculate relative expression. The sequences of primers were listed in Table 1.

**Table 1:** Primers sequences used for qPCR

Name		Primer sequence (5′–3′)
circTBX5(hsa_circ_0003176)	Forward	AATGTCAAGAATGCAAAGAGCAG
	Reverse	CTTTGATTCCCTCCATGCCCT
MyD88	Forward	GCATATGCCTGAGCGTTTCG
	Reverse	GTGGCCTTCTAGCCAACCTC
miR-146b-3p	Forward	GATTAGGCCCTGTGGACTCA
	Reverse	CTCAACTGGTGTCGTGGAGTC
miR-558	Forward	CTCCGAGTGAGCTGCTGTAC
	Reverse	TCAACTGGTGTCGTGGAGTC
miR-637	Forward	TTTAGACTGGGGGCTTTCGGG
	Reverse	CTCAACTGGTGTCGTGGAGTCG
miR-645	Forward	GATTCCGAGTCTAGGCTGGTAC
	Reverse	TCAACTGGTGTCGTGGAGTC
GAPDH	Forward	CAAATTCCATGGCACCGTCA
	Reverse	GACTCCACGACGTACTCAGC
U6	Forward	CTTCGGCAGCACATATACT
	Reverse	AAAATATGGAACGCTTCACG

### CircRNA identification

Divergent primers and convergent primers of circCARM1 were used to amplify circTBX5 and GAPDH from cDNA and gDNA of C28/I2 cells.

Total RNA was incubated with RNase R (2.5 U/µg; Epicentre, Madison, WI, USA). After digestion for 15 min at 37 °C conditions, total RNA was used for subsequent qPCR assay to detect the expression of circ_DHRS3 and GAPDH.

### Subcellular location of circRNA

Cytoplasmic and nuclear RNA were isolated using Cytoplasmic & Nuclear RNA Purification Kit (NGB-21000; Norgen Biotek, Thorold, Canada). An equal amount of cytoplasmic RNA (using GAPDH as a control) or nuclear RNA (using U6 as a control) was used for qPCR assay to determine the expression of circTBX5.

### CCK-8 assay

The treated cells with various transfections were cultured in 96-well plates, with three replications. Cells were then cultured for the indicated time, including 0 h, 24 h, 48 h and 72 h. Cells at different time points were treated with CCK-8 reagent (Beyotime) for 2 h. Cell viability was assessed according to optical density (OD) value at 450 nm detected by a microplate reader (Prolong, Beijing, China).

### EdU assay

To assess cell proliferative capacity, EdU assay was conducted using a Cell-Light EdU Apollo567 in Vitro Kit (Ribobio) according to the protocol. Briefly, cells after EdU treatment were fixed with formaldehyde and then washed with TBS. Cell nucleus was counterstained with DAPI. The number of EdU-positive cells was determined under a fluorescence microscope (Olympus, Tokyo, Japan).

### Western blot assay

Proteins were extracted using RIPA lysis buffer (Beyotime) and quantified by BCA Kit (Beyotime).The separated proteins were transferred onto PVDF membranes (Bio-Rad, Hercules, CA, USA). After incubation with skim milk, the membranes were exposed to the primary antibodies, all purchased from Abcam (Cambridge, MA, USA), including anti-Collagen II (ab188570), anti-Aggrecan (ab36861), anti-MMP13 (ab247309), anti-ADAMTS5 (ab41037), anti-MyD88 (ab133739), anti-IkBα (ab76429), anti-p-IkBα (phosphorylated IkBα; ab133462), anti-p65 (ab32536), anti-GAPDH (ab9485) and anti-Lamin B (ab16048). The membranes were subsequently probed with the second antibody (ab205718; Abcam). Finally, the protein blots were emerged using the ECL reagent (Beyotime).

### Flow cytometry assay

Cells were collected at 48 h post-transfection, trypsinized and washed with PBS. Then, cells were suspended in Annexin V-FITC binding buffer from the Annexin V-FITC Apoptosis Detection Kit (Beyotime) [23]. Next, cells were treated with Annexin V-FITC and propidium iodide (PI) according to the instructions. Cell apoptosis was monitored by a FACScan flow cytometer (BD Bioscience; San Jose, CA, USA).

### ELISA

Commercial kits, including Prostaglandin E2 (PGE2) Competitive ELISA Kit and Human IL-6 ELISA Kit, were bought from MultiSciences (Hangzhou, China). The procedures of ELISA were performed in accordance with respective specifications.

### RIP assay

RIP assay was used to verify whether circTBX5 interacted with Ago2, using IgG as a control. Simply put, C28/I2 cells were transfected with si-circTBX5 or si-NC and cultured for 48 h. Then, cells were collected and lysed by RIP lysis buffer from a RIP Kit (BersinBio, Guangzhou, China). Cell lysate was incubated with anti-Ago2 or anti-IgG at 4 °C overnight. The balanced protein A/G beads were added into IP samples and co-cultured for 1 h. After washing and eluting, RNA was isolated and used for qPCR assay.

### Pull-down assay

Biotin-labeled circTBX5 probe and oligo probe were provided by Geneseed. Streptavidin magnetic beads (BersinBio) were washed and incubated with circTBX5 probe or oligo probe to prepare probe-beads compounds [24]. Cell lysates were incubated with probe-beads compounds at room temperature for 2 h. After washing, RNA complex on beads was eluted and isolated for qPCR assay.

### Dual-luciferase reporter assay

The binding sites between circTBX5 and miR-558 were predicted by circBank (http://www.circbank.cn/) and circular RNA Interactome (https://circinteractome.nia.nih.gov/). The mutant sequence of circTBX5 (mut-circTBX5) was designed based on its wild-type sequence (wt-circTBX5). Then, reporter plasmids of mut-circTBX5 and wt-circTBX5 were constructed using pmirGLO plasmid. The binding site between miR-558 and MyD88 3′UTR was predicted by Targetscan (wt-circTBX5). Likewise, reporter plasmids of mut-MyD88 3′UTR and wt-MyD88 3′UTR were also constructed. C28/I2 cells were co-transfected with miR-588 (using miR-NC as a control) and wt or mut reporter plasmid of circTBX5 or MyD88 3′UTR. After culturing for 48 h, luciferase activity in cells was examined using the Dual-Luciferase Reporter Assay System (Promega, Madison, WI, USA).

### Statistical analysis

All experiments were independently conducted three times. Data were then processed using GraphPad Prism (version7.0; GraphPad, La Jolla, CA, USA) and shown as the mean ± standard deviation. The normality of data was examined using Shapiro–Wilk test. Difference comparisons between two groups were analyzed by Student’s *t*-test. Analysis of variance was conducted to evaluate the differences among multiple groups. Statistical significance was affirmed when *P* value was less than 0.05.

## Results

### CircTBX5 was highly expressed in OA cartilage tissues and IL-1β-treated C28/I2 cells

**Fig. 1:**
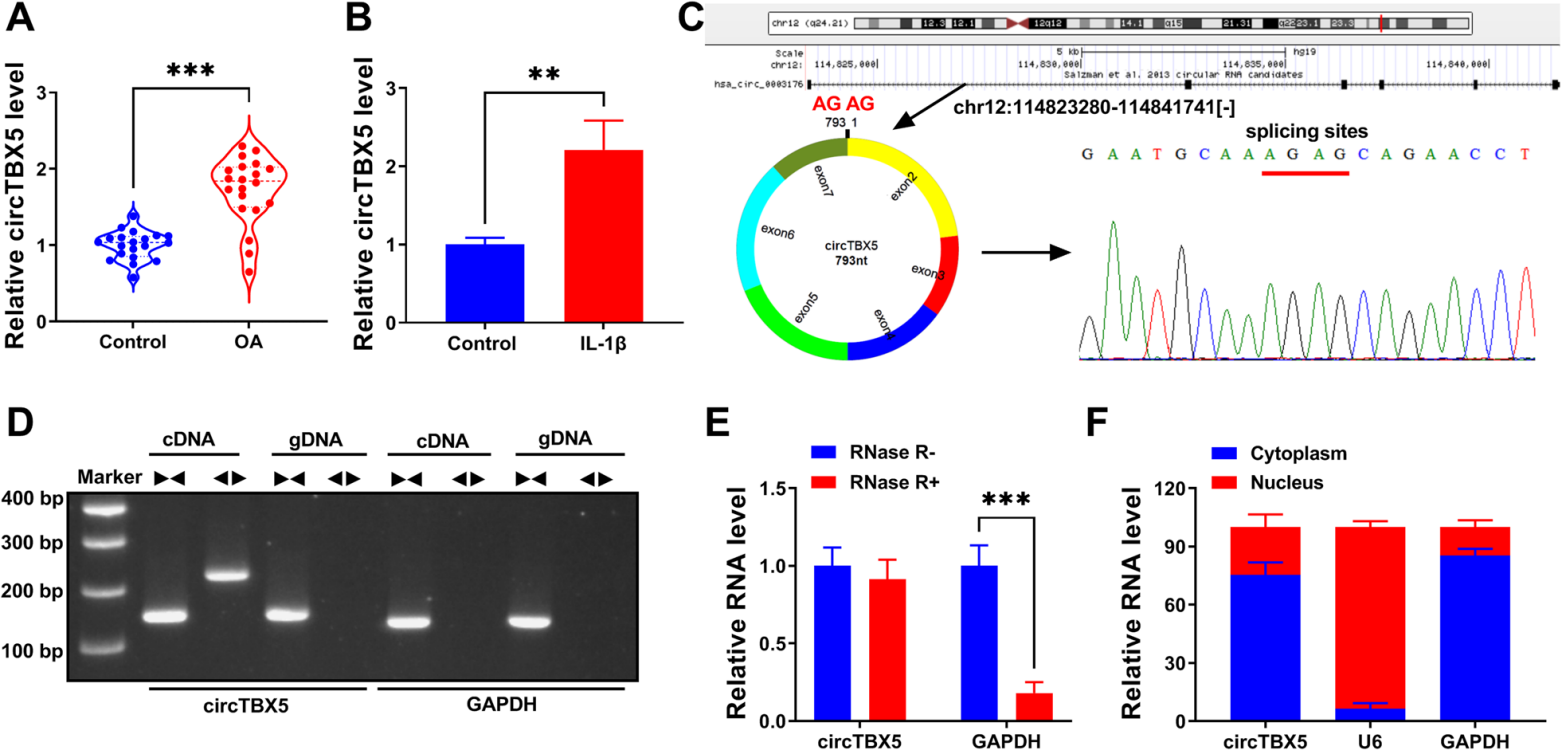
Upregulation of circTBX5 was shown in OA cartilage tissues and IL-1β-treated C28/I2 cells. **A** CircTBX5 expression in OA and normal cartilage tissues was checked by qPCR. **B** CircTBX5 expression in IL-1β-treated C28/I2 cells was checked by qPCR. **C** The formation of circTBX5 from its parental gene, TBX5. **D** CircTBX5 was identified using divergent primers. **E** The existence and stability of circTBX5 were identified using RNase R. **F** The location of circTBX5 in cytoplasm or in nucleus. **P < 0.01; ***P < 0.001

The expression and characteristics of circTBX5 were identified at first. As shown in Fig. 1A,B, the expression of circTBX5 was markedly increased in OA cartilage tissues relative to control group, as well as in IL-1β-treated C28/I2 cells relative to control group. Figure 1C clearly displayed that circTBX5 was produced by back-splicing at the exon2, exon3, exon4, exon5, exon6 and exon7 of TBX5 gene, with 793 nucleotides in length. To ensure the existence of this circRNA, we used divergent primers and convergent primers to amplify. The PCR product of circTBX5 was only obtained from cDNA by divergent primers (Fig. 1D). Besides, RNase R can digest all linear RNA molecules but hardly digest circRNA. We found that the expression of circTBX5 was rarely degraded by RNase R, compared to linear GAPDH (Fig. 1E). In addition, we characterized that circTBX5 was abundantly expressed in the cytoplasm but rarely expressed in the nucleus (Fig. 1F). These results verified the existence of circTBX5, determined its location and clarified its expression in OA conditions.

### CircTBX5 downregulation relieved IL-1β-induced C28/I2 cell proliferative impairment, ECM degradation, apoptosis and inflammation

**Fig. 2:**
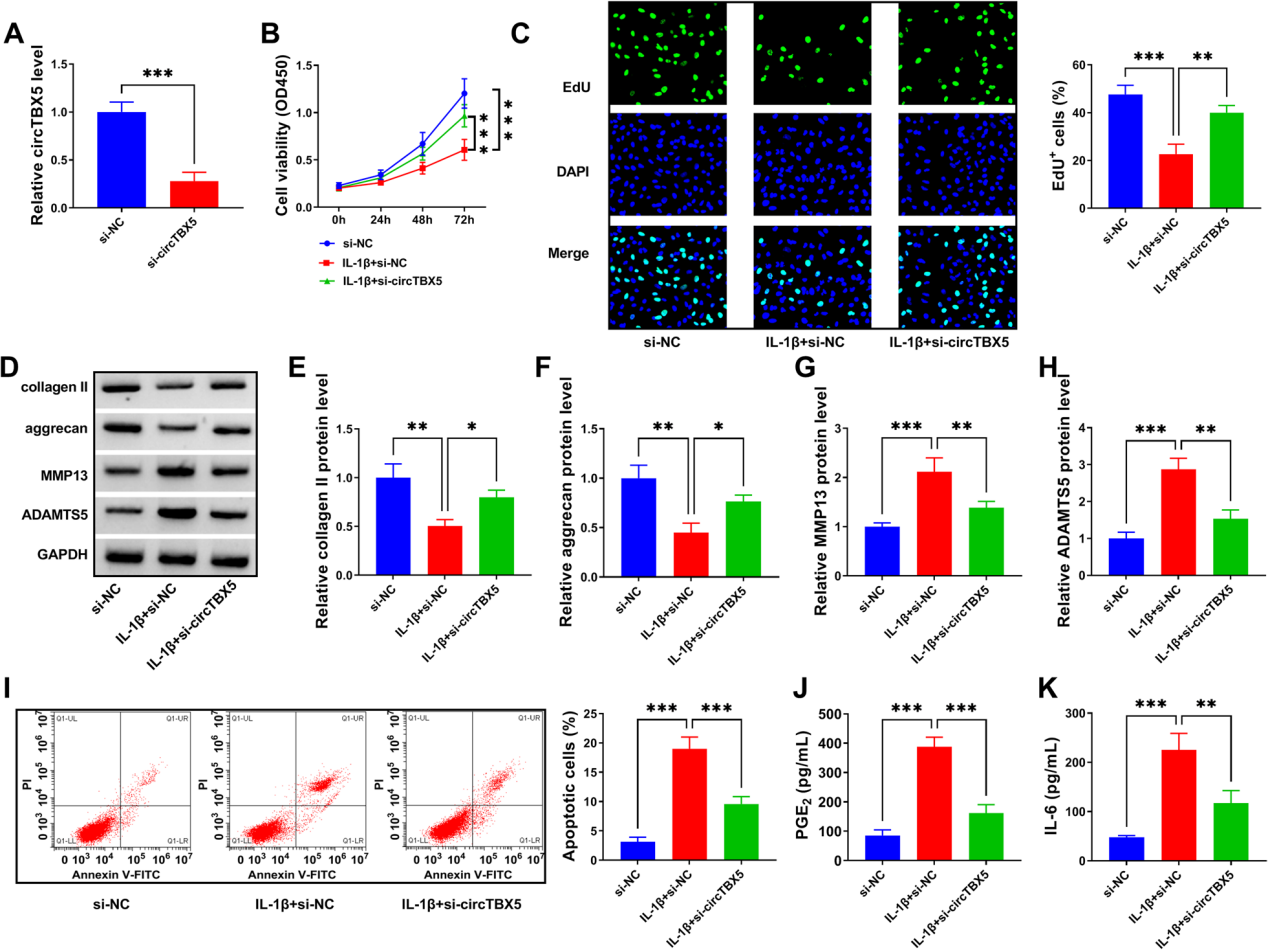
CircTBX5 knockdown alleviated IL-1β-induced C28/I2 cell injury. **A** The interference efficiency of si-circTBX5 on circTBX5 expression was determined by qPCR assay. **B** Cell viability in IL-1β-treated C28/I2 transfected with si-circTBX5 was detected by CCK-8 assay. **C** Cell proliferation in IL-1β-treated C28/I2 transfected with si-circTBX5 was detected by EdU assay. **D**–**H** The expression levels of collagen II, aggrecan, MMP13 and ADAMTS5 in IL-1β-treated C28/I2 transfected with si-circTBX5 were detected by western blot assay. **I** Cell apoptosis in IL-1β-treated C28/I2 transfected with si-circTBX5 was detected by flow cytometry. **J**, **K** The release of PGE_2_ and IL-6 in IL-1β-treated C28/I2 transfected with si-circTBX5 was detected by ELISA. *P < 0.05; **P < 0.01; ***P < 0.001

The use of si-circTBX5 remarkably reduced the expression level of circTBX5 in C28/I2 cells (Fig. 2A). In function, knockdown of circTBX5 restored IL-1β-depleted C28/I2 cell viability by CCK-8 assay (Fig. 2B). The data from EdU assay displayed that cell proliferative capacity was impaired by IL-1β, while circTBX5 knockdown largely promoted cell proliferation (Fig. 2C). The protein levels of collagen II and aggrecan were enhanced by circTBX5 downregulation in IL-1β-treated C28/I2 cells, and the protein levels of MMP13 and ADAMTS5 were repressed by circTBX5 downregulation in IL-1β-treated C28/I2 cells (Fig. 2D–H). In addition, IL-1β-induced C28/I2 cell apoptosis was considerably alleviated by circTBX5 knockdown (Fig. 2I). Moreover, we monitored that the release of inflammatory mediators (PGE_2_ and IL-6) was stimulated by the treatment of IL-1β, while further circTBX5 knockdown attenuated the release of them (Fig. 2J–K). Overall, IL-1β-induced C28/I2 cell proliferative impairment, ECM degradation, apoptosis and inflammation were alleviated by circTBX5 knockdown.

### CircTBX5 directly bound to miR-558

**Fig. 3:**
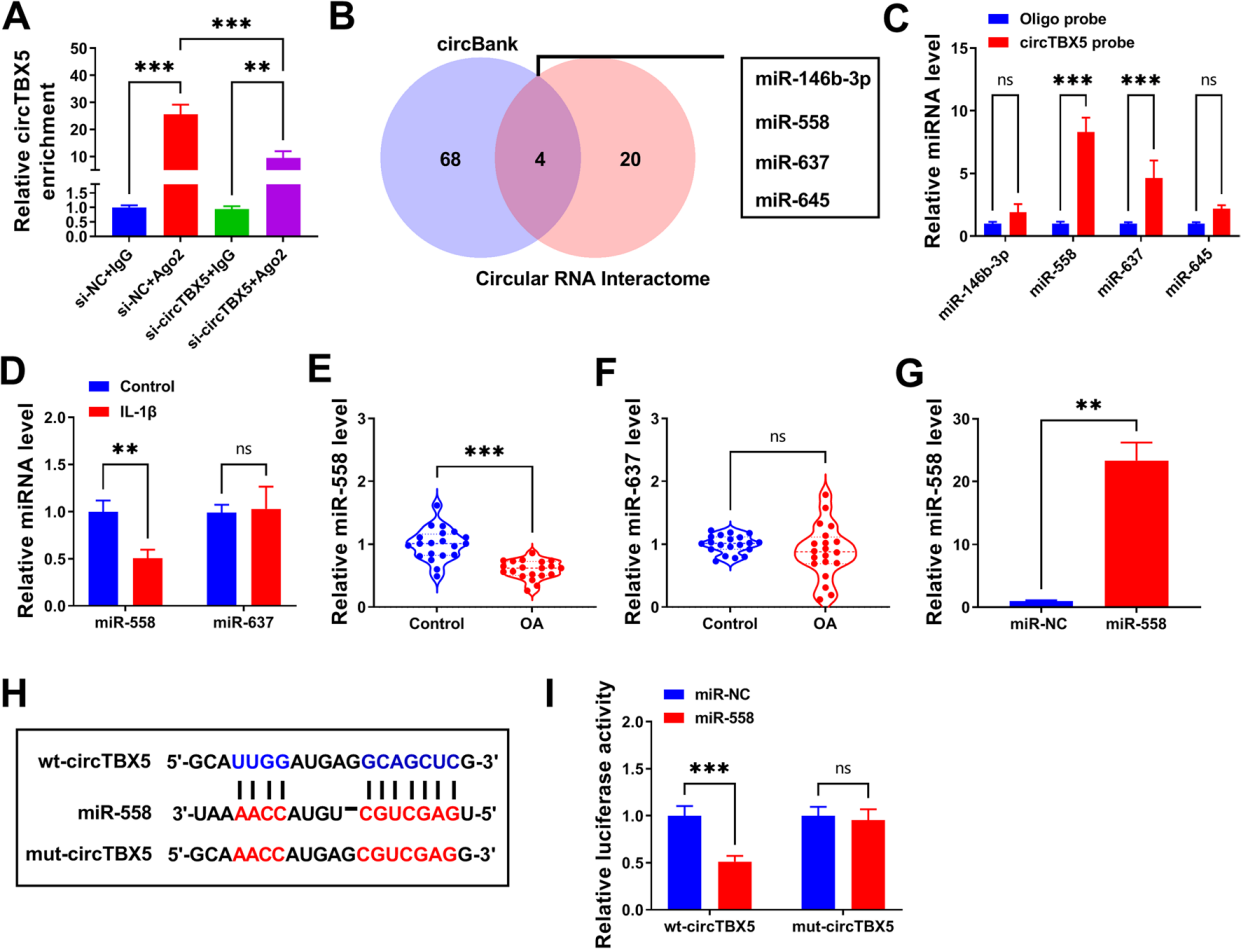
MiR-588 was targeted by circTBX5. **A** RIP was performed to determine whether circTBX5 could be enriched by Ago2. **B** Circular RNA interactome and circBank were used to predict miRNAs targeted by circTBX5. **C** Pull-down assay was performed to screen miRNAs bound to circTBX5. **D** The expression levels of miR-588 and miR-637 in IL-1β-treated C28/I2 cells were measured by qPCR. **E**, **F** The expression levels of miR-588 and miR-637 in OA and normal cartilage tissues were checked by qPCR. **G** The efficiency of miR-558 mimic was checked by qPCR. **H**, **I** The binding between circTBX5 and miR-558 was validated by dual-luciferase reporter assay. **P < 0.01; ***P < 0.001; ns: no significance

Through RIP assay, we found that circTBX5 was enriched by Ago2 relative to IgG, and it was less enriched by Ago2 in C28/I2 cells transfected with si-circTBX5 compared with that in cells transfected with si-NC (Fig. 3A), suggesting that circTBX5 might interact with target miRNAs through Ago2-mediated manner. Subsequently, circBank and Circular RNA Interactome were utilized to predict miRNAs targeted by circTBX5, and a total of 4 miRNAs were commonly predicted by two databases (Fig. 3B). Pull-down assay was next conducted to screen these miRNAs. The data displayed that only miR-588 and miR-637 could be abundantly pulled down by circTBX5 probe (Fig. 3C). Moreover, miR-558 showed lower expression level in IL-1β-treated C28/I2 cells and OA cartilage tissues, while miR-637 expression showed no significance (Fig. 3D–F). MiR-558 was selected for further verification. As shown in Fig. 3G, the transfection of miR-558 markedly strengthened the expression of miR-558 in C28/I2 cells. For dual-luciferase reporter assay, the constructs of wt-circTBX5 and mut-circTBX5 were shown in Fig. 3H. The data presented that the enhanced expression of miR-558 strikingly impaired luciferase activity in C28/I2 cells transfected with wt-circTBX5 but not mut-circTBX5 (Fig. 3I), strongly verifying the binding between circTBX5 and miR-558.

### *CircTBX5 downregulation relieved IL-1β-induced C28/I2 cell injury *via* upregulating miR-558*

**Fig. 4:**
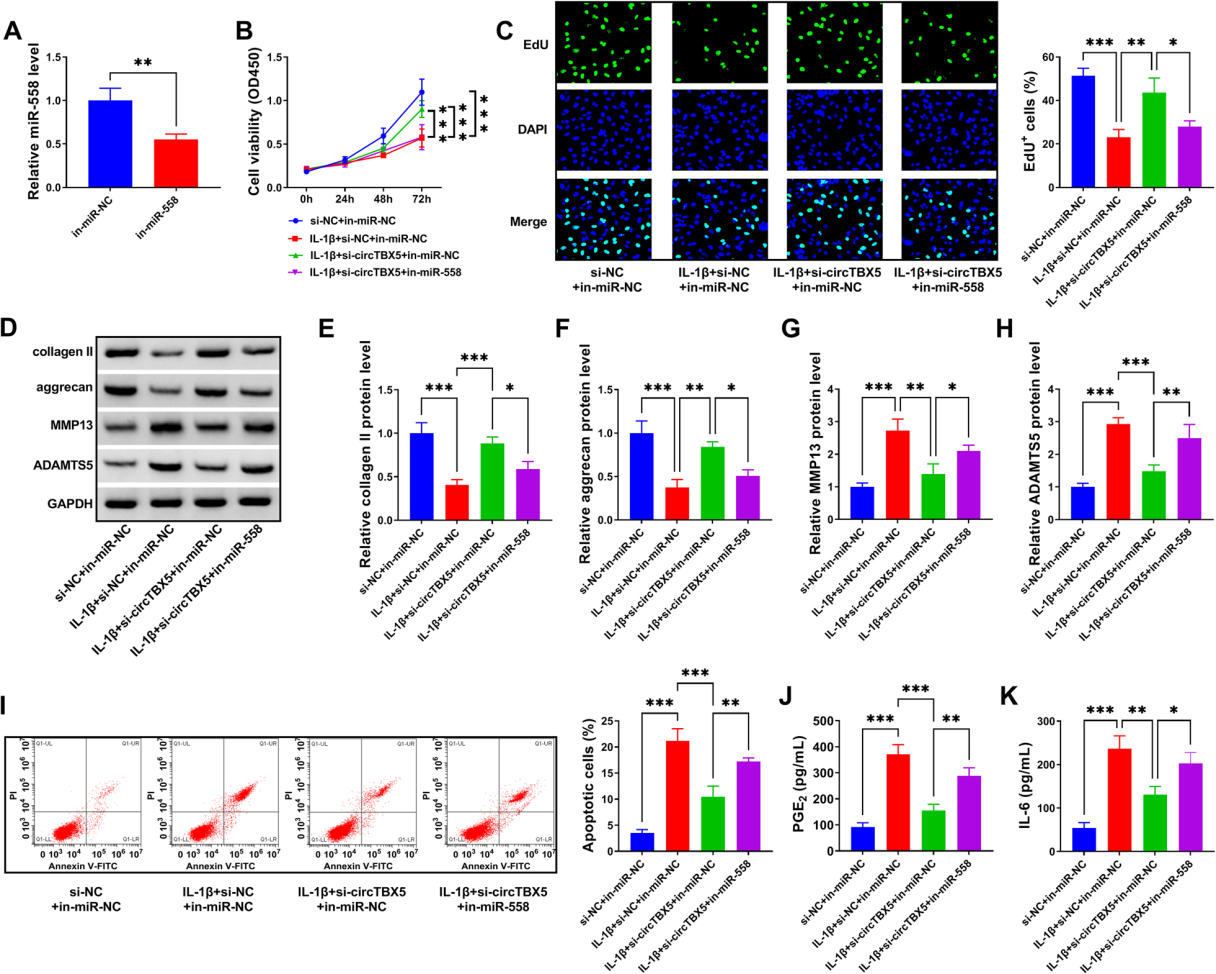
CircTBX5 knockdown alleviated IL-1β-induced C28/I2 cell injury by enriching miR-558. **A** The efficiency of in-miR-558 was checked by qPCR. In IL-1β-treated C28/I2 cells with circTBX5 knockdown or combined circTBX5 knockdown and miR-558 inhibition, **B** cell viability was monitored by CCK-8 assay. **C** Cell proliferation was monitored by EdU assay. **D**–**H** The protein levels of collagen II, aggrecan, MMP13 and ADAMTS5 were monitored by western blot. **I** Cell apoptosis was monitored by flow cytometry assay. **J**, **K** The release of PGE_2_ and IL-6 was monitored by ELISA. *P < 0.05; **P < 0.01; ***P < 0.001

To determine whether circTBX5 relieved IL-1β-induced C28/I2 cell injury by targeting miR-558, rescue experiments were performed. The transfection of in-miR-558 in C28/I2 cells significantly decreased the expression of miR-558 (Fig. 4A). In function, circTBX5 knockdown-rescued cell viability and cell proliferative capacity in IL-1β-treated C28/I2 cells were substantially repressed by the introduction of in-miR-558 (Fig. 4B, C). The expression levels of collagen II and aggrecan in IL-1β-treated C28/I2 cells were recovered by circTBX5 knockdown, while their expression levels were partially depleted by the transfection of in-miR-588 relative to in-miR-NC (Fig. 4D–F). The expression levels of MMP13 and ADAMTS5 were repressed by circTBX5 knockdown, while additional in-miR-588 transfection largely restored their expression levels (Fig. 4D, G, H). Additionally, IL-1β-stimulated C28/I2 cell apoptosis was suppressed by circTBX5 knockdown, while the suppression was largely relieved by additional miR-558 inhibition (Fig. 4I). The release of PGE_2_ and IL-6 in IL-1β-treated C28/I2 cells was effectively blocked by circTBX5 downregulation, while the release of PGE_2_ and IL-6 was notably recovered by miR-558 repression (Fig. 4J, K). The data suggested that miR-558 repression reversed the role of circTBX5 knockdown, verifying that circTBX5 knockdown relieved IL-1β-induced C28/I2 cell injury via upregulating miR-558.

### MiR-558 targeted MyD88

**Fig. 5:**
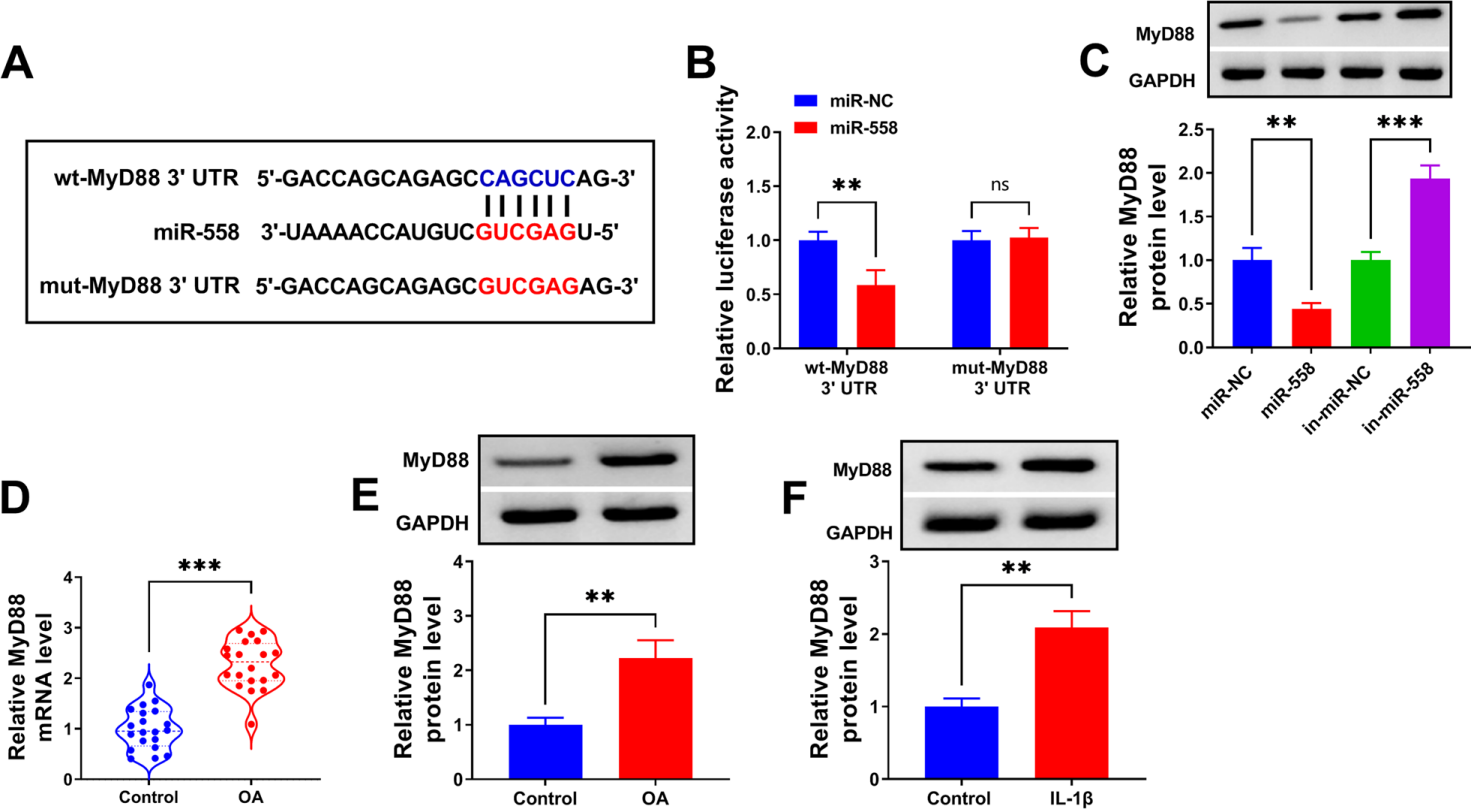
MyD88 was a target of miR-558. **A**, **B** The binding between miR-558 and MyD88 3′UTR was verified by dual-luciferase reporter assay. **C** The expression of MyD88 in C28/I2 cells with miR-558 enrichment or miR-558 inhibition was detected by western blot. **D**, **E** The expression of MyD88 in OA and normal cartilage tissues was checked by qPCR and western blot. **F** The expression of MyD88 in IL-1β-treated C28/I2 cells was detected by western blot. **P < 0.01; ***P < 0.001

Among the target genes of miR-558 predicted by Targescan, we noticed that miR-558 harbored a special binding site on MyD88 3′UTR. The constructs of wt-MyD88 3′UTR and mut-MyD88 3′UTR were shown in Fig. 5A. The data from dual-luciferase reporter assay evidenced that miR-558 overexpression strikingly diminished luciferase activity in C28/I2 cells transfected with wt-MyD88 3′UTR rather than mut-MyD88 3′UTR (Fig. 5B). In addition, we observed that the expression of MyD88 protein was notably decreased in C28/I2 cells transfected with miR-558 but notably strengthened in C28/I2 cells transfected with in-miR-558 (Fig. 5C), suggesting that miR-558 negatively regulated MyD88 expression. The expression of MyD88 (at mRNA and protein levels) was significantly elevated in OA cartilage tissues relative to control (Fig. 5D, E). MyD88 expression was also enhanced in IL-1β-treated C28/I2 cells (Fig. 5F). The data manifested that MyD88 showed an opposite expression pattern with miR-558, and miR-558 negatively regulated its expression.

### MiR-558 upregulation attenuated IL-1β-induced C28/I2 cell injury by sequestering MyD88

**Fig. 6:**
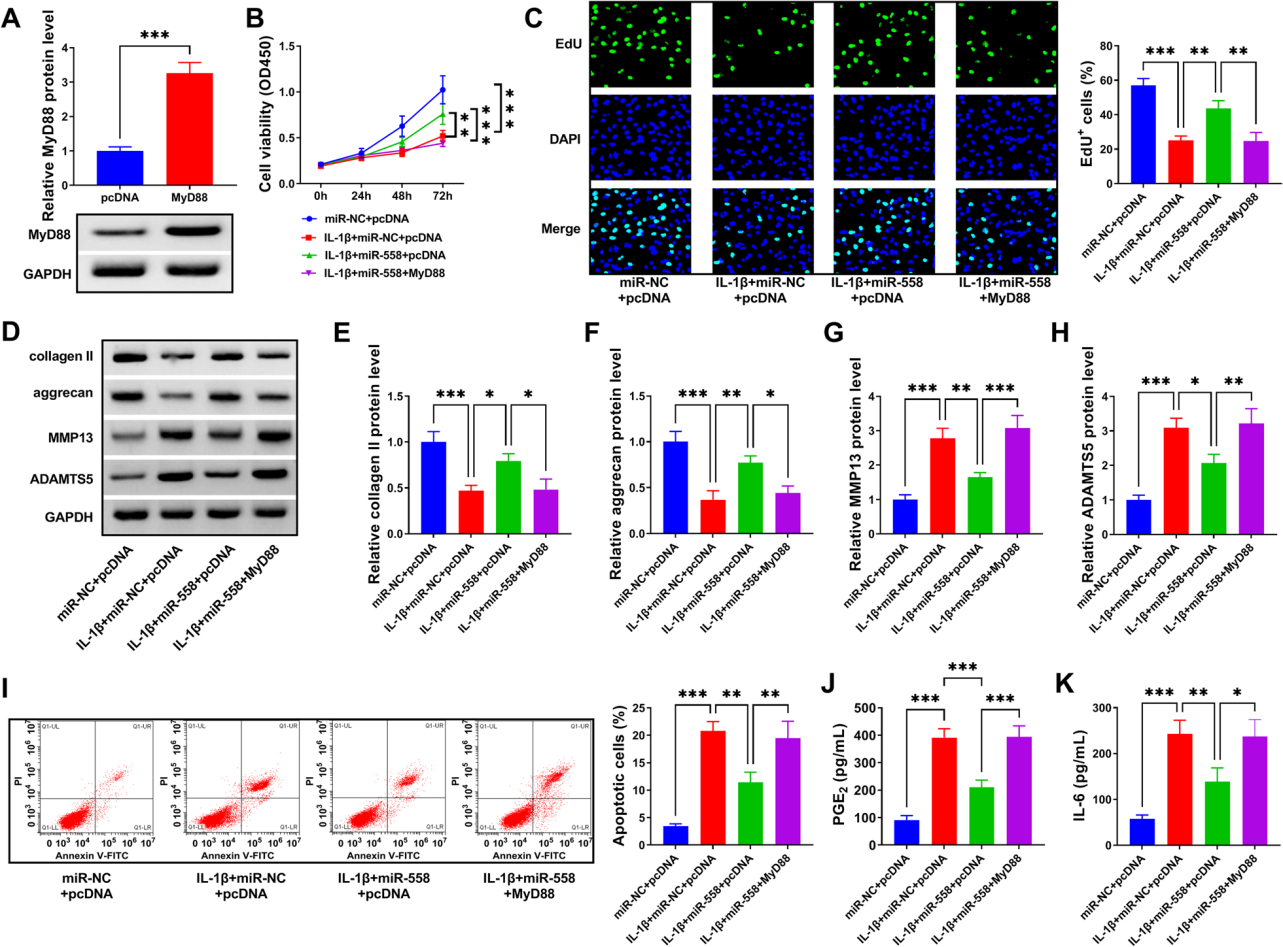
MiR-558 enrichment alleviated IL-1β-induced C28/I2 cell injury by suppressing MyD88. **A** The efficiency of MyD88 overexpression was checked by western blot. In IL-1β-treated C28/I2 cells with miR-558 enrichment or combined miR-558 enrichment and MyD88 overexpression, **B**, **C** cell viability and cell proliferation were determined by CCK-8 and EdU assay. **D**–**H** The protein levels of collagen II, aggrecan, MMP13 and ADAMTS5 were monitored by western blot. **I** Cell apoptosis was monitored by flow cytometry assay. **J**, **K** The release of PGE_2_ and IL-6 was monitored by ELISA. *P < 0.05; **P < 0.01; ***P < 0.001

To examine whether miR-588 was involved in IL-1β-induced C28/I2 cell injury by targeting MyD88, rescue experiments were further conducted. The application of MyD88 overexpression vector considerably reinforced the expression level of MyD88 in C28/I2 cells (Fig. 6A). In function, IL-1β-depleted cell viability and cell proliferation were largely restored by miR-558 upregulation, while MyD88 reintroduction effectively repressed cell viability and cell proliferation of IL-1β-treated C28/I2 cells (Fig. 6B, C). The protein levels of collagen II and aggrecan were promoted by miR-558 enrichment in IL-1β-treated C28/I2 cells, while overexpression of MyD88 largely impaired their expression levels (Fig. 6D–F). In contrast, the protein levels of MMP13 and ADAMTS5 were suppressed by miR-558 enrichment in IL-1β-treated C28/I2 cells, while overexpression of MyD88 largely recovered their expression levels (Fig. 6D, G, H). In addition, IL-1β-stimulated C28/I2 cell apoptosis was substantially alleviated by miR-558 upregulation, while MyD88 reintroduction enhanced the apoptosis rate of C28/I2 cells (Fig. 6I). The release of PGE_2_ and IL-6 aggravated by IL-1β was largely alleviated by miR-558 upregulation, while additional MyD88 overexpression promoted the release of PGE_2_ and IL-6 (Fig. 6J, K). The data evidenced that miR-558 enrichment prevented IL-1β-induced C28/I2 cell injury by depleting MyD88.

### CircTBX5 targeted miR-558 to regulate MyD88 expression and NF-кB signaling pathway

**Fig. 7:**
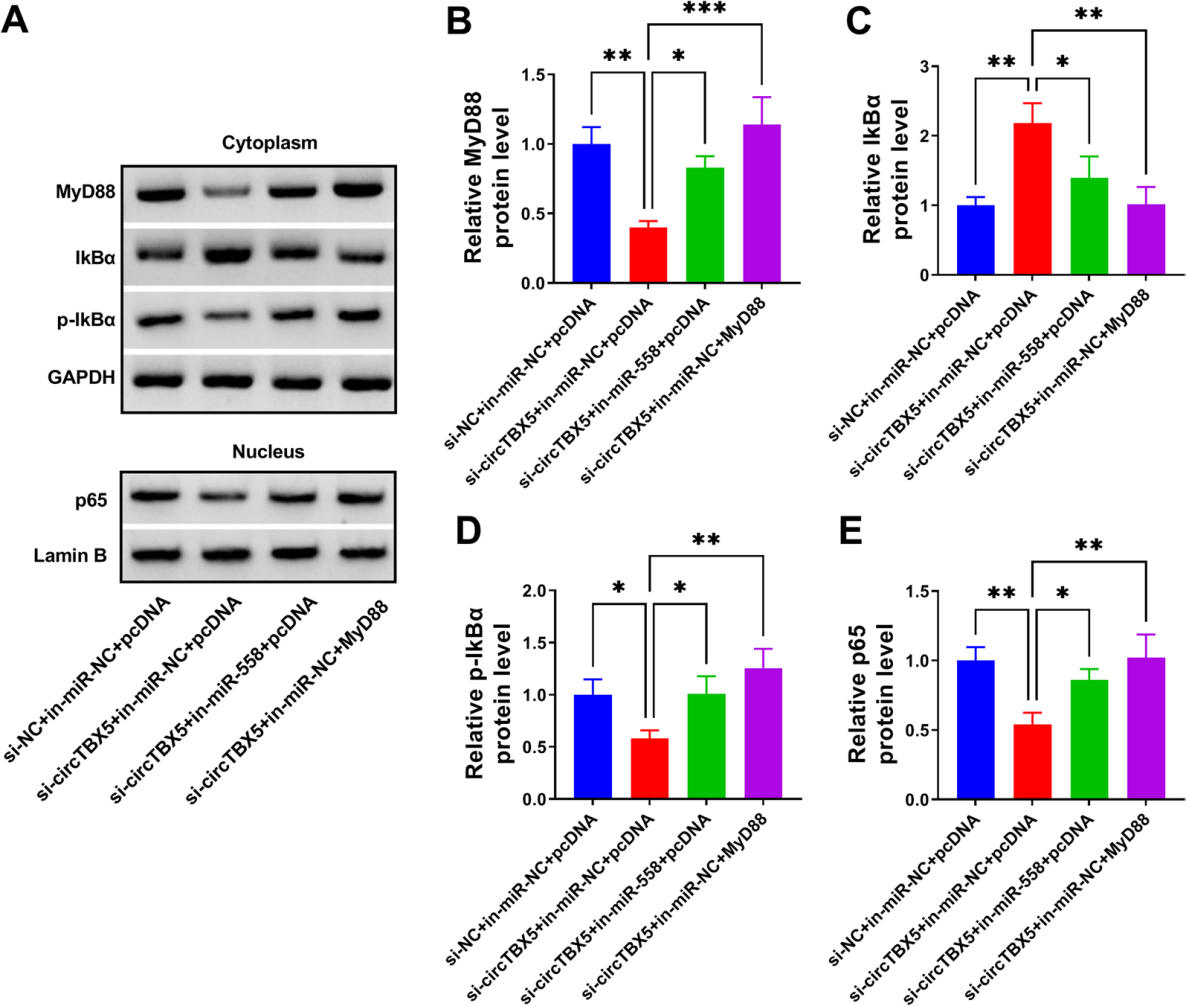
CircTBX5 targeted miR-558 to regulate MyD88 expression and NF-кB signaling pathway. In C28/I2 cells transfected with si-NC + in-miR-NC + pcDNA, si-circTBX5 + in-miR-NC + pcDNA, si-circTBX5 + in-miR-558 + pcDNA or si-circTBX5 + in-miR-NC + MyD88, **A**–**E** the protein levels of MyD88, IkBα, p-IkBα and P65 were measured by western blot. *P < 0.05; **P < 0.01; ***P < 0.001

Nuclear protein and cytoplasmic protein were separately extracted from C28/I2 cells transfected with si-NC + in-miR-NC + pcDNA, si-circTBX5 + in-miR-NC + pcDNA, si-circTBX5 + in-miR-558 + pcDNA or si-circTBX5 + in-miR-NC + MyD88. Herein, the expression levels of MyD88, IkBα and p-IkBα were examined in cytoplasmic protein, with GAPDH as an internal reference. The expression level of p65 was checked in nuclear protein, with Lamin B as an internal reference. As a result, the expression levels of MyD88, p-IkBα and p65 were markedly decreased in C28/I2 cells by si-circTBX5 + in-miR-NC + pcDNA but largely restored by si-circTBX5 + in-miR-558 + pcDNA or si-circTBX5 + in-miR-NC + MyD88 (Fig. 7A, B, D, E). In contrast, the expression level of IkBα was markedly reinforced in C28/I2 cells by si-circTBX5 + in-miR-NC + pcDNA but largely repressed by si-circTBX5 + in-miR-558 + pcDNA or si-circTBX5 + in-miR-NC + MyD88 (Fig. 7A, C). These results uncovered that circTBX5 knockdown-suppressed MyD88 expression and NF-кB signaling activity were recovered by miR-558 depletion or MyD88 overexpression, indicating that circTBX5 targeted miR-558 to regulate MyD88 expression, thus modulating the NF-кB signaling pathway. To confirm our results, pyrrolidine dithiocarbamate (PDTC), an inhibitor of inflammation and NF-кB activation, was used here. The transfection of OE-circTBX5 could notably increase circTBX5 expression in C28/I2 cells (Additional file 1: Fig. S1A). Functionally, cell viability and proliferation reduced by IL-1β were largely decreased by circTBX5 overexpression, while the addition of PDTC recovered cell viability and proliferation (Additional file 1: Fig. S1B, C). The protein levels of collagen II and aggrecan repressed by IL-1β were further reduced by circTBX5 overexpression, while PDTC effectively restored their levels (Additional file 1: Fig. S1D–F). The protein levels of MMP13 and ADAMTS5 strengthened by IL-1β were further enhanced by circTBX5 overexpression, while PDTC effectively impaired their levels (Additional file 1: Fig. S1D, G, H). Moreover, circTBX5 overexpression strengthened IL-1β-induced cell apoptosis, while PDTC addition alleviated cell apoptotic rate (Additional file 1: Fig. S1I). The release of PGE_2_ and IL-6 aggravated by IL-1β was largely reinforced by circTBX5 overexpression, while the use of PDTC repressed the release of PGE_2_ and IL-6 (Additional file 1: Fig. S1J, K). Overall, circTBX5 regulated NF-кB signaling pathway in IL-1β-induced C28/I2 cells.

## Discussion

This was a preliminary study to disclose the potential role of circTBX5 in OA. First, the upregulation of circTBX5 was observed in OA cartilage tissues and IL-1β-treated C28/I2 cells. Second, IL-1β-induced C28/I2 cell proliferative impairment, ECM degradation, apoptosis and inflammation were attenuated by circTBX5 knockdown. Third, circTBX5 targeted miR-558 to positively regulate the expression of MyD88. Fourth, circTBX5 knockdown impaired the activity of NF-кB signaling. All these findings elucidated the role of circTBX5 in IL-1β-induced C28/I2 dysfunction and exploited the regulatory network of circTBX5 to partly explain the functional mechanism of circTBX5.

Numerous circRNAs were demonstrated to regulate cell viability and proliferation in IL-1β-treated chondrocytes. For example, circ_0094742 overexpression rescued IL-1β-depleted cell viability [25], while circ_0114876 downregulation promoted cell viability and growth in IL-1β-treated chondrocytes [15]. Our study presented that IL-1β-impaired cell viability and proliferative capacity were largely restored by circTBX5 knockdown. In OA development, the turnover of ECM macromolecules is destroyed by catabolic changes, leading to the secretion of inflammatory mediators and the loss of proteoglycans, fibrillar and non-fibrillar collagens, principally including collagen type II and aggrecan [6, 26]. Besides, matrix metalloproteinases and ADAMTSs play a considerable role in OA via degrading collagen type II and aggrecan, and they have been proposed to be therapeutic targets for OA [27]. In our study, we monitored that IL-1β treatment diminished the expression of collagen II and aggrecan but strengthened the expression of MMP13 and ADAMTS5, which was consistent with previous studies [28–30]. In addition, the dysregulation of apoptosis is a landmark event in various pathological states, such as developmental anomalies and degenerative diseases [31]. The degeneration and apoptosis of chondrocytes are the leading cause of OA [32]. Here, the treatment of IL-1β strikingly induced C28/I2 cell apoptosis, while circTBX5 knockdown substantially prevented IL-1β-induced apoptosis. Moreover, it was widely clarified that IL-1β administration stimulated the release of proinflammatory mediators in chondrocytes, mainly including IL-6, TNF-α and PGE_2_ [33, 34]. Consistent with these results, we monitored that the release of IL-6 and PGE_2_ was strongly promoted in IL-1β-treated C28/I2 cells, while knockdown of circTBX5 largely repressed the release of IL-6 and PGE_2_. Collectively, our study reported that IL-1β-induced C28/I2 cell injuries, including cell viability and proliferation impairment, ECM degradation, cell apoptosis and inflammation production, were largely suppressed by circTBX5 downregulation, hinting that the protective effect of circTBX5 repression on chondrocytes may be an effective approach for OA treatment in clinical practice.

After a preliminary understanding of the role of circTBX5 in chondrocytes, we attempted to explore its functional mechanism. MiR-558 was screened and validated to be a target of circTBX5. It was previously determined to be downregulated in OA chondrocytes after IL-1β stimulation [35]. Afterwards, miR-558 was also shown to be downregulated in OA cartilage, and IL-1β-activated MMP1 and MMP13 expression was inhibited by miR-558 enrichment [19]. Consistent with the results, our study verified that the expression of miR-558 was strikingly lower in OA cartilage tissues and IL-1β-treated C28/I2 cells. Acting as a target of circTBX5, inhibition of miR-558 reversed the effects of circTBX5 knockdown, leading to the restoration of cell viability and proliferation impairment, ECM degradation, cell apoptosis and inflammation production. In contrast, overexpression of miR-558 effectively restrained IL-1β-induced C28/I2 cell dysfunction. We proposed that circTBX5 knockdown alleviated chondrocyte injury via strengthening miR-558 expression.

Further work identified MyD88 as a downstream target of miR-558, and circTBX5 knockdown impaired the expression of MyD88 via enriching miR-558. MyD88 is a key downstream adapter protein of numerous Toll-like regulators (TLRs) and IL-1 receptors and plays a key role in signal transduction of downstream inflammatory factors [22]. MyD88 has been widely studied to be involved in OA. For example, MyD88 expression was declined in OA cartilage tissues and chondrocytes induced with IL-1β, and MyD88 silencing reduced the level of inflammation and blocked matrix degradation [36, 37]. In agreement with these studies, significant upregulation of MyD88 was also shown in OA cartilage tissues and IL-1β-administered chondrocytes in our study. In function, MyD88 overexpression reversed the effects of miR-558 enrichment, thus recovering IL-1β-induced C28/I2 cell dysfunction. TLR signaling induces the activation of NF-κB signaling, which controls an array of inflammatory responses [38]. Whereupon, the TLR/MyD88/NF-κB signaling pathway plays a crucial role in multiple inflammatory diseases [39–41]. In chondrocytes, IL-1β stimulated the secretion of inflammatory factors via activating the NF-κB signaling, while MyD88 silencing weakened the activity of NF-κB signaling [36]. Given that circTBX5 regulated IL-1β-induced chondrocyte dysfunction, we guessed that circTBX5 might modulate the NF-κB signaling and checked the levels of NF-κB signaling pathway proteins (IkBα and p65). The data of western blot assay displayed that the expression levels of phosphorylated IkBα and p65 were notably weakened by circTBX5 knockdown, while additional miR-558 inhibition or MyD88 overexpression largely enhancing their expression levels, strongly evidencing that circTBX5 knockdown governed the miR-558/MyD88 axis to repressing the activity of NF-κB signaling. However, these studies were mainly conducted in human cells in vitro, which couldn’t predict the results in vivo. Our work only observed the potential role of circTBX5/miR-558/MyD88 axis in cell models of OA, and its role in vivo should be further studied in animal models to start translational research.

## Conclusion

Overall, circTBX5 showed high expression in OA cartilage tissues and IL-1β-treated C28/I2 cells. Knockdown of circTBX5 attenuated IL-1β-induced C28/I2 cell proliferative arrest, ECM degradation, apoptosis and inflammatory response. We proposed that circTBX5 governed the miR-558/MyD88 axis to regulate the activity of NF-κB signaling and thereby regulate IL-1β-induced C28/I2 cell dysfunction. The management of circTBX5/miR-558/MyD88 axis may be a promising strategy for OA treatment. However, further study is required to verify the functional effects of circTBX5 in vivo.

## Supplementary Information

**Additional file 1: Figure S1.** CircTBX5 regulated NF-кB signaling pathway to regulate IL-1β-induced C28/I2 cell injury. **A** The efficiency of circTBX5 overexpression was ensured. **B**–**K** IL-1β-treated C28/I2 cells were transfected with OE-circTBX5 or vector, and IL-1β-treated C28/I2 cells were transfected with OE-circTBX5 and then treated with PDTC. **B**, **C** cell viability and cell proliferation were determined by CCK-8 and EdU assay. **D**–**H** The protein levels of collagen II, aggrecan, MMP13 and ADAMTS5 were monitored by western blot. **I** Cell apoptosis was monitored by flow cytometry assay. **J**, **K** The release of PGE2 and IL-6 was monitored by ELISA. *P < 0.05; **P < 0.01; ***P < 0.001.
