# Supplementary figures and images for: CircTBX5 knockdown modulates the miR-558/MyD88 axis to alleviate IL-1β-induced inflammation, apoptosis and extracellular matrix degradation in chondrocytes via inactivating the NF-κB signaling
Source: J Orthop Surg Res. 2023 Jul 1;18:477. doi: 10.1186/s13018-023-03949-5 (PMC10314403; doi:10.1186/s13018-023-03949-5)

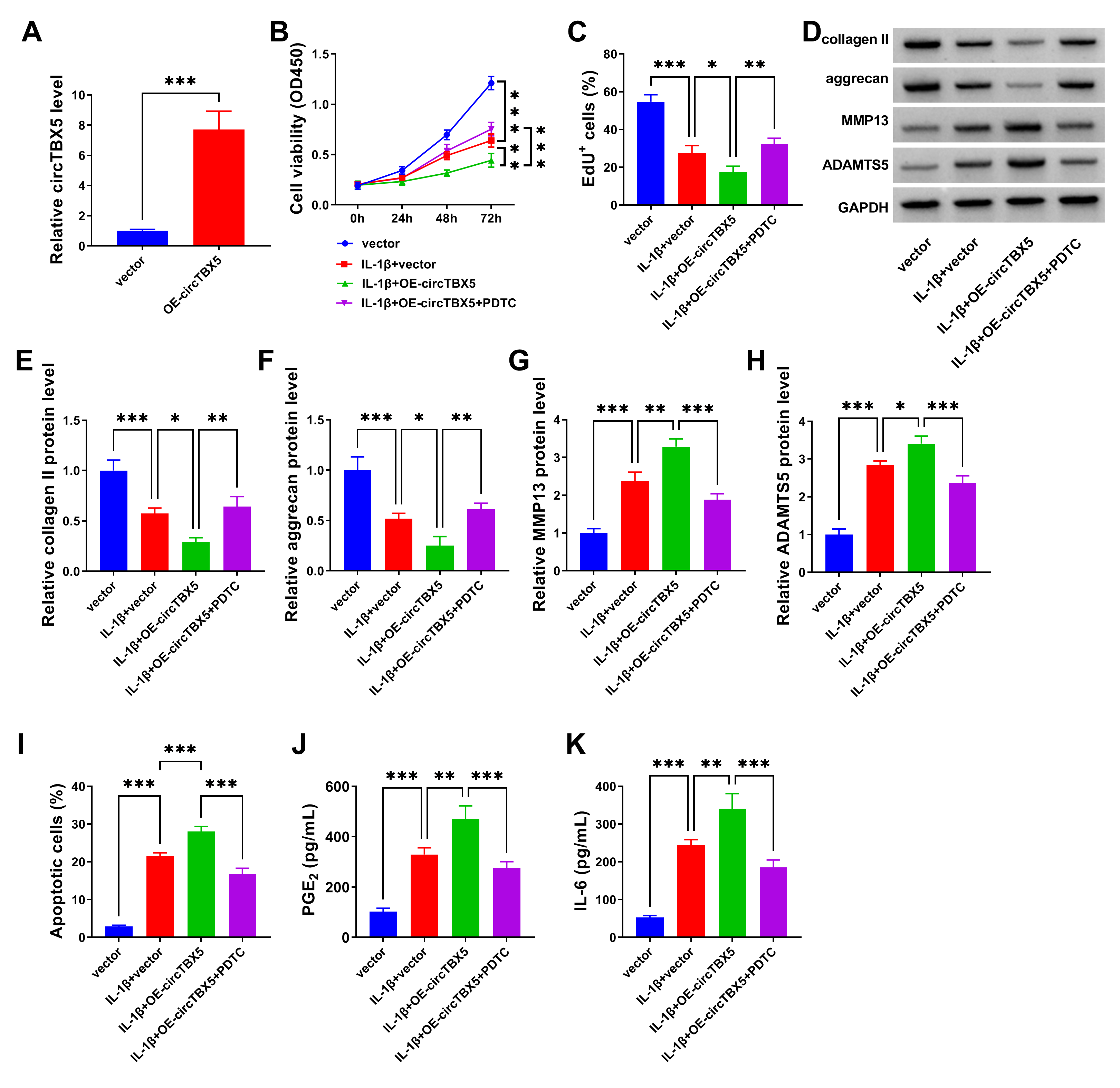

Supplement: Supplementary file 1 — Additional file 1: Figure S1. CircTBX5 regulated NF-кB signaling pathway to regulate IL-1β-induced C28/I2 cell injury. A The efficiency of circTBX5 overexpression was ensured. B–K IL-1β-treated C28/I2 cells were transfected with OE-circTBX5 or vector, and IL-1β-treated C28/I2 cells were transfected with OE-circTBX5 and then treated with PDTC. B, C cell viability and cell proliferation were determined by CCK-8 and EdU assay. D–H The protein levels of collagen II, aggrecan, MMP13 and ADAMTS5 were monitored by western blot. I Cell apoptosis was monitored by flow cytometry assay. J, K The release of PGE2 and IL-6 was monitored by ELISA. *P < 0.05; **P < 0.01; ***P < 0.001. [file 13018_2023_3949_MOESM1_ESM.tif]
